# Supplementary material for: Wireworm (Coleoptera: Elateridae) genomic analysis reveals putative cryptic species, population structure, and adaptation to pest control
Source: Commun Biol. 2020 Sep 7;3:489. doi: 10.1038/s42003-020-01169-9 (PMC7477237; doi:10.1038/s42003-020-01169-9)
Supplement: Supplementary file 1 — Supplementary Information [file 42003_2020_1169_MOESM1_ESM.pdf]

Supplemental Figures

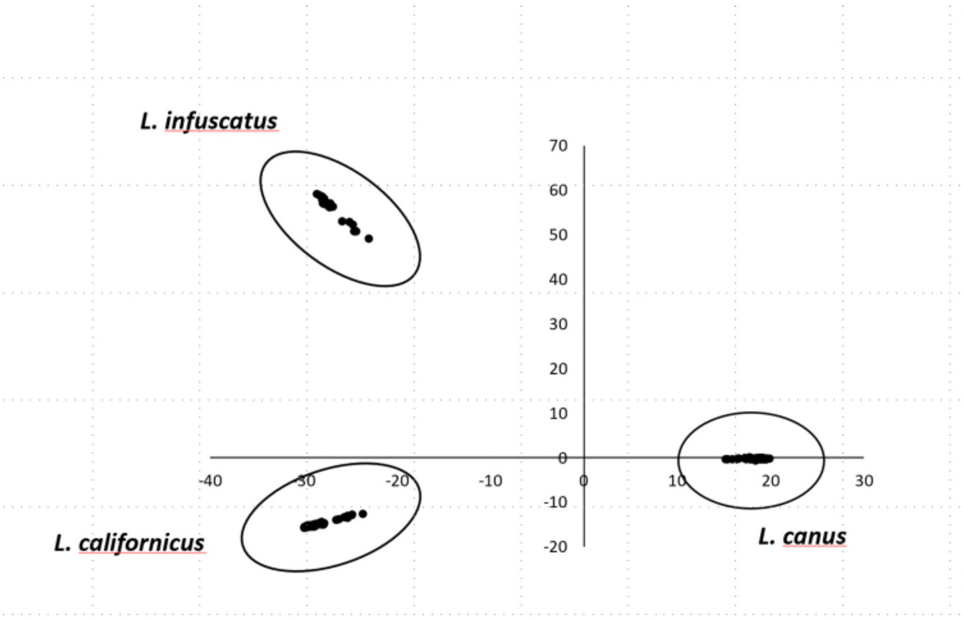

Supplementary Figure 1. PCA of the full RADseq dataset with three species (*L. californicus*, *L. infuscatus*, and *L. canus*). The first axis accounted for 49.5% of the genetic variation, and the second axis accounted for 32.0% of the genetic variation. Here *L. canus* separated on the first axis, whereas this species separated on the second axis for PCA conducted after subsampling to equal sample sizes per species (see Fig. 2); this difference in results was driven by unequal sizes in the full dataset, with the large sample size of *L. canus* causing this species to separate more strongly.

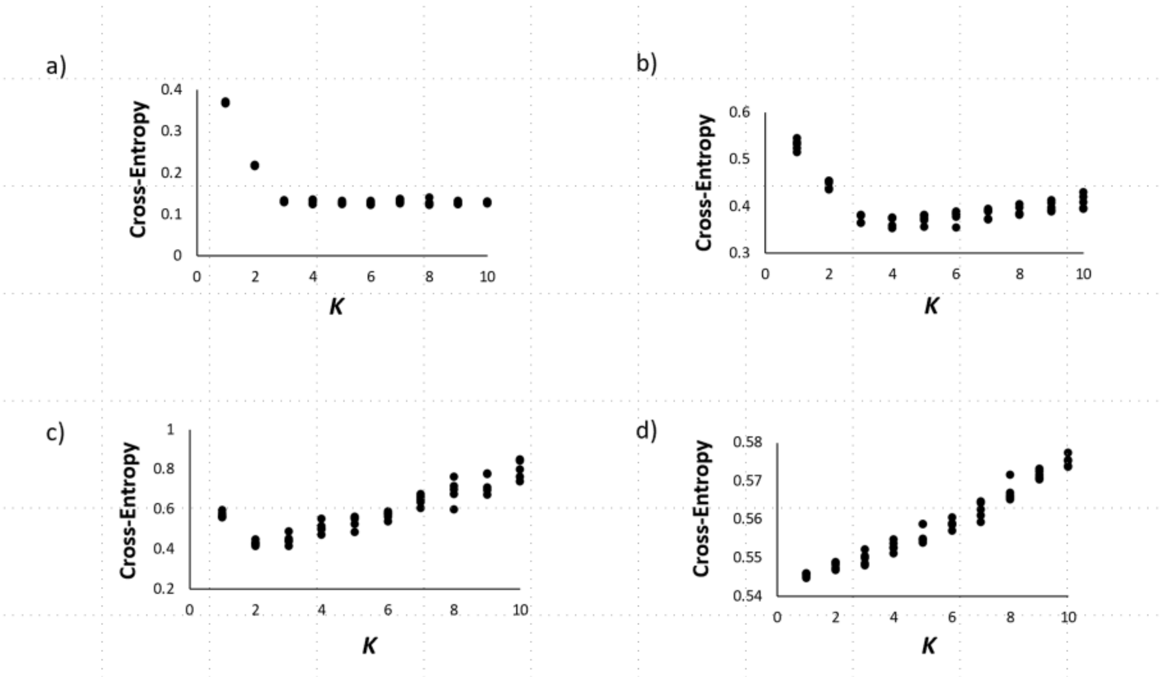

Supplementary Figure 2. Cross-entropy values from sNMF analysis for *K*=1 through *K* =10 for (a) all samples; (b) *L. californicus*; (c) *L. infuscatus*; (d) *L. canus*.

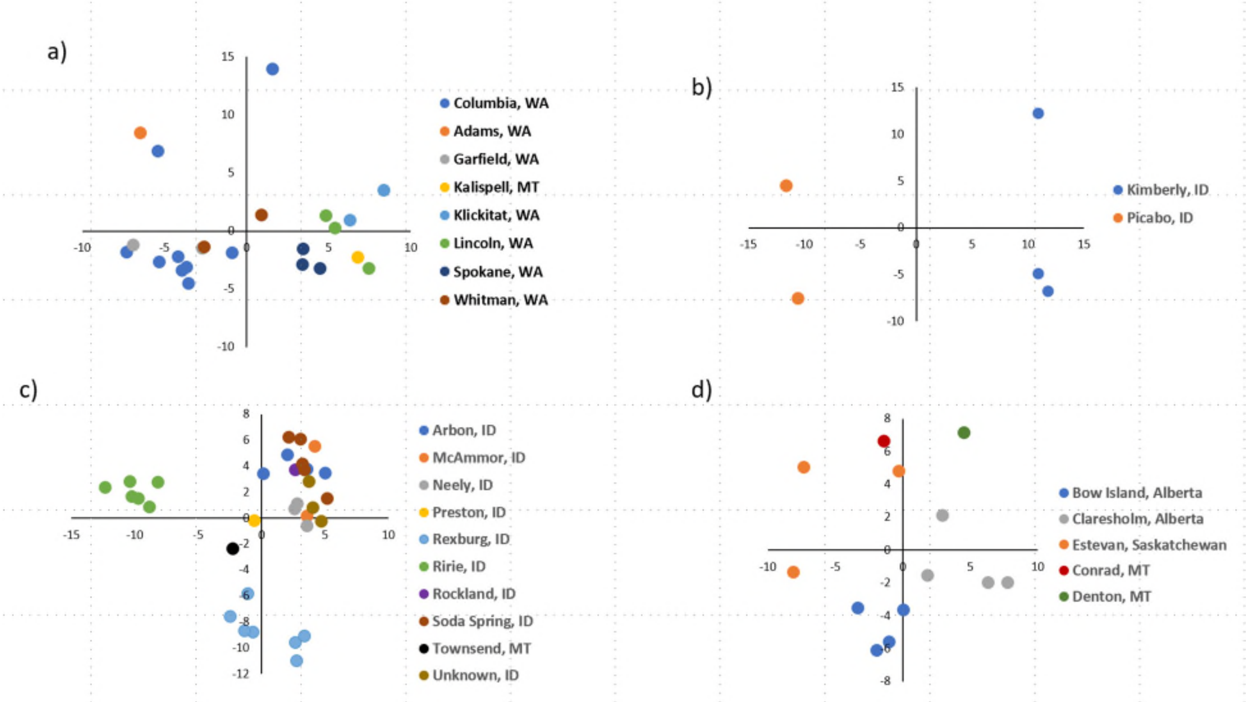

Supplementary Figure 3. PCA for *L. californicus* groups, including a) Group A; first and second axes account for 9.84% and 7.31% of genetic variation; b) Group B; first and second axes account for 42.1% and 17.0% of genetic variation; c) Group C; first and second axes account for 8.52% and 7.83% of genetic variation; d) Group D; first and second axes account for 14.1% and 13.2% of genetic variation.

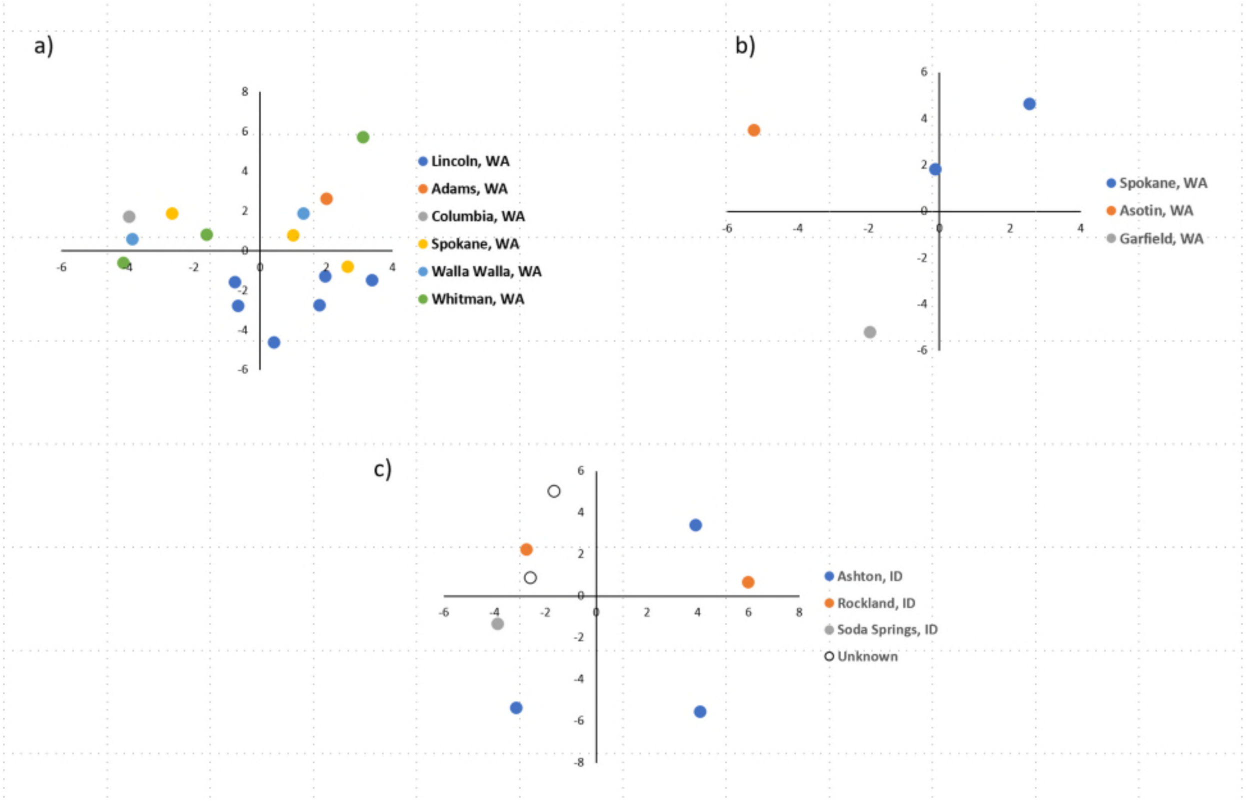

Supplementary Figure 4. PCA for *L. infuscatus* groups, including a) Group E; first and second axes account for 11.1% and 10.5% of genetic variation; b) Group F; first and second axes account for 46.3% and 33.3% of genetic variation; c) Group G; first and second axes account for 19.7% and 18.8% of genetic variation.

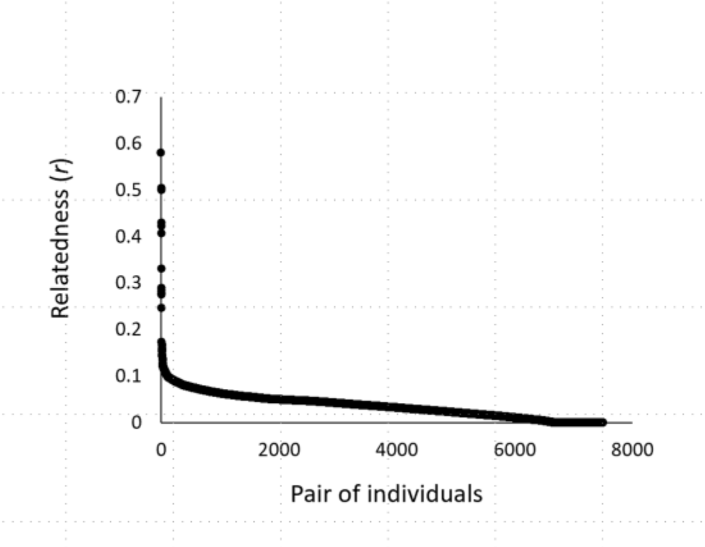

Supplementary Figure 5. Relatedness values for each pair of *L. canus* individuals sampled from Hermiston, Oregon, arranged from the highest to the lowest values from left to right.

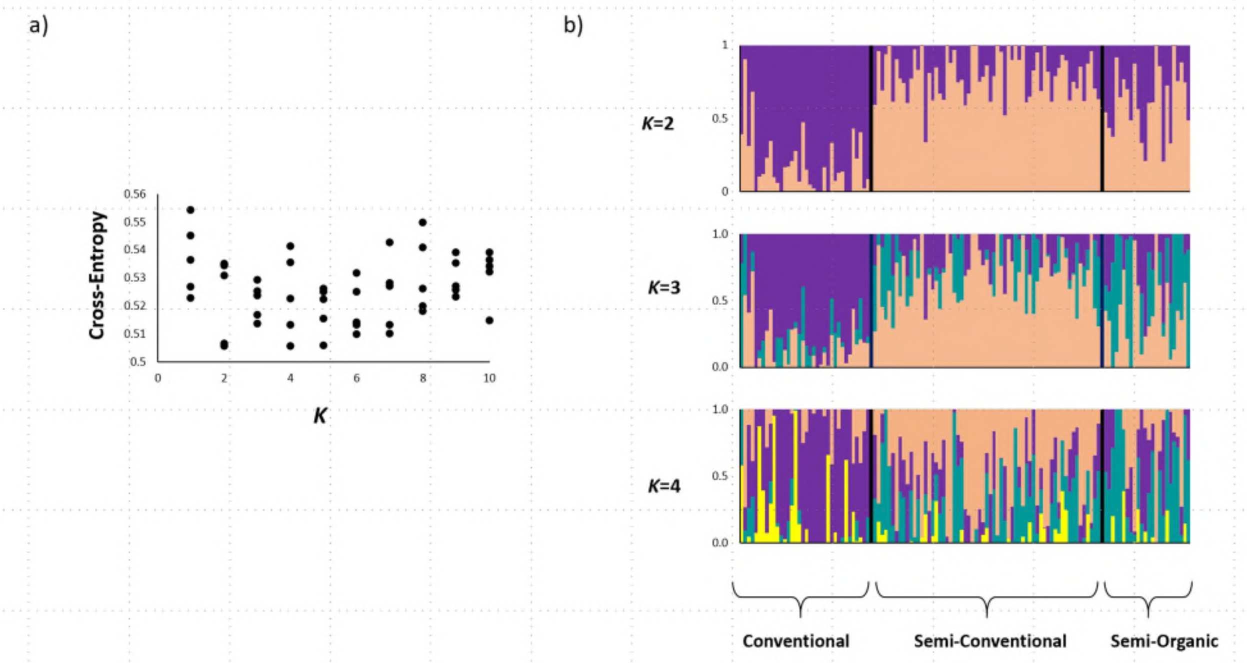

Supplementary Figure 6. sNMF analysis for Hermisont, Oregon *L. canus* using only SNPs identified as F_{ST} outliers. a) Cross-entropy values from sNMF analysis for K=1 through K =10. b) Ancestry proportions for the run with the lowest cross-entropy value for each of K=2-4.

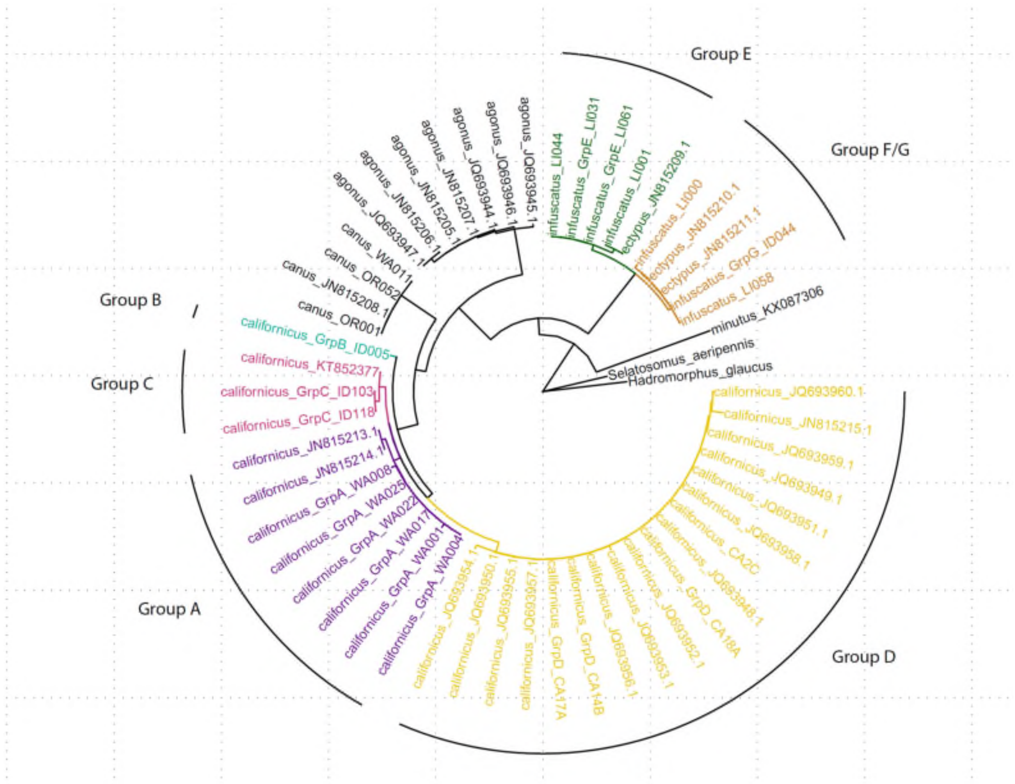

Figure 7. Neighbor Joining tree of 279bp *16S rDNA* sequences from this study (*Limonius* samples and two outgroups, *Hadromorphus glaucus* and *Selatosomus aeripennis*) and from *Limonius* samples retrieved from GenBank from other studies. For sequences from this study, sample names are included, along with the RADseq group if RADseq data were collected for the sample. Sample names for sequences from other studies include Genbank accession numbers.

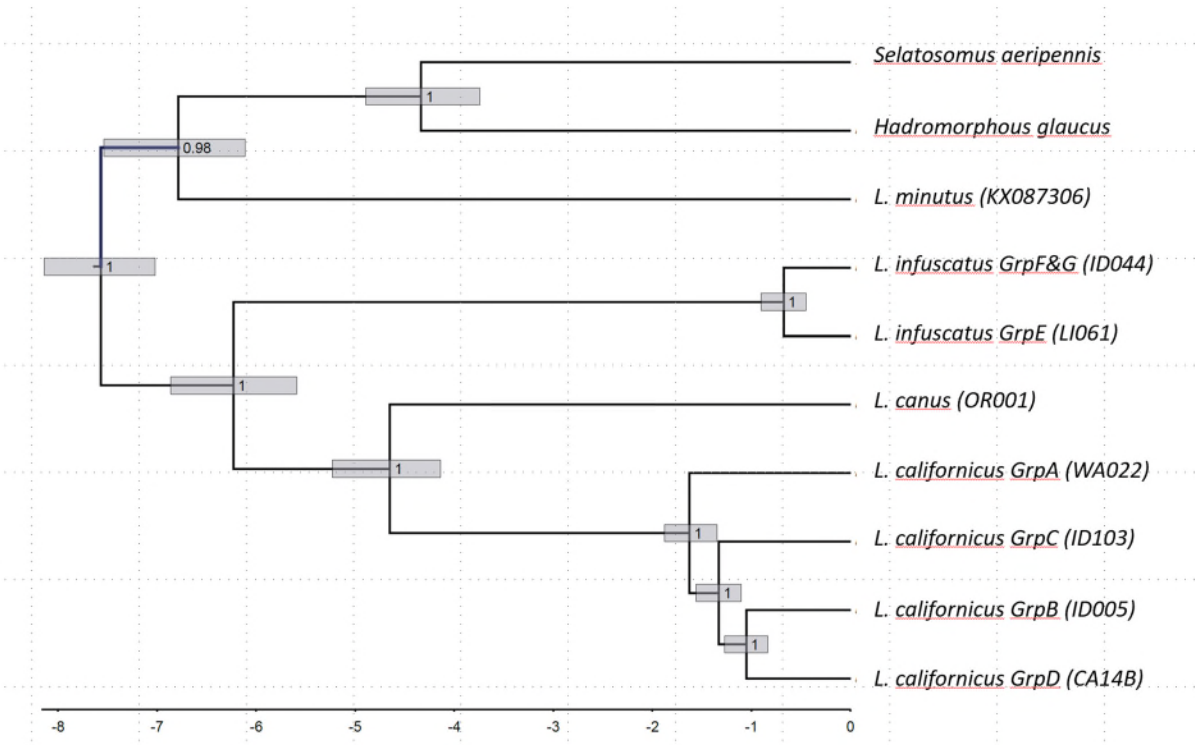

Figure 8. Maximum clade credibility tree generated using BEAST2 with full-length COI sequences, using a strict molecular clock and a between-lineage divergence rate of 3.54% per million years. Nodes show posterior probabilities, and node bars show 95% highest posterior density (HPD) intervals for divergence times. Scale is in millions of years.

Supplemental Tables

Supplementary Table 1. Relatedness (*r*), pesticide treatment, and sample dates for highly related pairs of *L. canus* individuals from agricultural plots with different types of pesticide treatment from Hermiston, Oregon. The number of days between sampling of individuals in each pair is also reported. Pairs are ordered from the highest to lowest relatedness values.

**Sample 1**			**Sample 2**			
**Pesticide treatment**	**Sample date**	**Sample**	**Pesticide treatment**	**Sample date**	***r***	**Days between sampling**
Semi-Conventional	8/7/2014	OR084	Semi-Conventional	8/14/2014	0.579	7
Conventional	8/14/2014	OR120	Conventional	8/7/2014	0.504	7
Semi-Conventional	8/21/2014	OR070	Semi-Conventional	8/7/2014	0.499	14
Semi-Conventional	8/14/2014	OR071	Semi-Conventional	8/14/2014	0.43	0
Semi-Conventional	8/14/2014	OR081	Semi-Conventional	8/14/2014	0.422	0
Conventional	6/27/2014	OR137	Conventional	9/10/2014	0.406	75
Semi-Conventional	8/14/2014	OR078	Semi-Conventional	8/14/2014	0.33	0
Semi-organic	6/9/2014	OR171	Semi-Conventional	4/7/2015	0.289	302
Semi-Conventional	3/27/2015	OR151	Semi-Conventional	3/27/2015	0.284	0
Semi-Conventional	8/7/2014	OR073	Semi-Conventional	8/14/2014	0.277	7
Semi-Conventional	8/7/2014	OR078	Semi-Conventional	8/14/2014	0.274	7
Semi-Conventional	4/7/2015	OR163	Semi-Conventional	4/7/2015	0.246	0

Supplemental Results

Phylogenetic analysis: Neighbor Joining trees

For both our samples and the GenBank samples, all samples collected from the same geographic region fell into the same mtDNA lineage on the Neighbor Joining trees (Fig. 7, Fig. S6). For *L. californicus*, COI sequences retrieved from GenBank were from the same geographic locations as our study except one sample from Aberdeen, Idaho, which is geographically close to our southeastern Idaho sample sites. The 16S sequences retrieved from GenBank for *L. californicus* fell into lineages corresponding with RADseq Groups A and D. The GenBank Group A samples were collected further north than our Group A samples in British Columbia (Oliver), indicating that the geographic range of Group A extends to this location. The Group D GenBank samples were collected relatively close to our samples in Alberta (Claresholm and Taber) and Saskatchewan (Drake, Moose Jaw, Swift Current, Wakaw).

For *L. infuscatus*, the COI GenBank Group E samples were collected east of our Group E samples, from northern Idaho (Boundary) and western Montana (Flathead and Granite), indicating that the geographic range of this lineage extends east of our sample sites. The GenBank Group G samples were collected from southwestern Montana (Gallatin), in between our Washington and Idaho sampling sites. We did not have mtDNA sequence data from Group F samples that also had RADseq data; however, RADseq data indicate Groups F and G are not highly divergent (Fig. 3, Fig. 4, Table 3). No 16S sequences were available on GenBank for *L. infuscatus*.

For both the COI and 16S trees, several *L. ectypus* sequences retrieved from GenBank fell within the *L. infuscatus* lineages, potentially indicating these individuals are actually the same species. Dhafer [1] has argued for uniting these two species based on morphological similarity, whereas Etzler [2] has argued that the two species are distinct, but that *L. ectypus* only occurs east of the central Great Plains. All *L. ectypus* sequences retrieved from GenBank for our study were collected west of the Great Plains, and therefore the genetic similarity identified here between *L. ectypus* and *L. infuscatus* does not necessarily contradict the conclusion of species distinction by Etzler. For COI GenBank samples, one *L. ectypus* sample collected in southwestern British Columbia (Victoria) fell within Group E, and one *L. ectypus* sample collected from southern Alberta (Lethbridge) fell within Group F/G (Fig. 7). For 16S GenBank samples, one *L. ectypus* sample from Fort Macleod, Alberta fell within Group E, and two samples from this same location fell within Group G (Fig S5).

1. Al Dhafer HM. Revision of the North American Species of Limonius (Coleoptera: Elateridae). Transactions of the American Entomological Society 2009;135**:**209-352.
2. Etzler FE: Identification of economic wireworms using traditional and molecular methods. Montana State University, 2013.
