# Supplementary material for: Wireworm (Coleoptera: Elateridae) genomic analysis reveals putative cryptic species, population structure, and adaptation to pest control
Source: Commun Biol. 2020 Sep 7;3:489. doi: 10.1038/s42003-020-01169-9 (PMC7477237; doi:10.1038/s42003-020-01169-9)
Supplement: Supplementary file 5 — Description of Additional Supplementary Files [file 42003_2020_1169_MOESM5_ESM.pdf]

Descriptions of Additional Supplementary Files

Supplementary Data 1. Sample collection metadata and inclusion in RADseq, COI sequencing, and 16S sequencing analyses (Y=sample included, N=sample not included).

Supplementary Data 2. Genomic positions and *p* values for SNPs identified as F_{ST} outliers for comparisons between agricultural plots in Hermiston, Oregon.

Supplementary Data 3. Maximum genetic distance along scaffolds ("Max distance") for F_{ST} outlier SNPs identified from comparisons between agricultural plots in Hermiston, Oregon. Boxes surround SNPs occurring on the same scaffold. This table only includes outlier SNPs occurring on scaffolds that had more than one outlier SNP. SNPs less than ~800bp apart are associated with the same restriction cut site.
