# Supplementary material for: Wireworm (Coleoptera: Elateridae) genomic analysis reveals putative cryptic species, population structure, and adaptation to pest control
Source: Commun Biol. 2020 Sep 7;3:489. doi: 10.1038/s42003-020-01169-9 (PMC7477237; doi:10.1038/s42003-020-01169-9)
Supplement: Supplementary file 6 — Reporting Summary [file 42003_2020_1169_MOESM6_ESM.pdf]

nature research

Corresponding author(s): Kimberly R Andrews

Last updated by author(s): Jan 16, 2020

Reporting Summary

Nature Research wishes to improve the reproducibility of the work that we publish. This form provides structure for consistency and transparency in reporting. For further information on Nature Research policies, see Authors & Referees and the Editorial Policy Checklist.

Statistics

For all statistical analyses, confirm that the following items are present in the figure legend, table legend, main text, or Methods section.

n/a	Confirmed	
☐	☒	The exact sample size (*n*) for each experimental group/condition, given as a discrete number and unit of measurement
☐	☒	A statement on whether measurements were taken from distinct samples or whether the same sample was measured repeatedly
☐	☒	The statistical test(s) used AND whether they are one- or two-sided<br>*Only common tests should be described solely by name; describe more complex techniques in the Methods section.*
☐	☒	A description of all covariates tested
☐	☒	A description of any assumptions or corrections, such as tests of normality and adjustment for multiple comparisons
☐	☒	A full description of the statistical parameters including central tendency (e.g. means) or other basic estimates (e.g. regression coefficient) AND variation (e.g. standard deviation) or associated estimates of uncertainty (e.g. confidence intervals)
☐	☒	For null hypothesis testing, the test statistic (e.g. *F*, *t*, *r*) with confidence intervals, effect sizes, degrees of freedom and *P* value noted<br>*Give P values as exact values whenever suitable.*
☐	☒	For Bayesian analysis, information on the choice of priors and Markov chain Monte Carlo settings
☐	☒	For hierarchical and complex designs, identification of the appropriate level for tests and full reporting of outcomes
☒	☐	Estimates of effect sizes (e.g. Cohen's *d*, Pearson's *r*), indicating how they were calculated

Our web collection on statistics for biologists contains articles on many of the points above.

Software and code

Policy information about availability of computer code

Data collection	No software was used to collect the data
Data analysis	Software names and version numbers are provided in the manuscript. We provide a link to a github repository containing detailed code we used for running the software.

For manuscripts utilizing custom algorithms or software that are central to the research but not yet described in published literature, software must be made available to editors/reviewers. We strongly encourage code deposition in a community repository (e.g. GitHub). See the Nature Research guidelines for submitting code & software for further information.

Data

Policy information about availability of data

All manuscripts must include a data availability statement. This statement should provide the following information, where applicable:

- Accession codes, unique identifiers, or web links for publicly available datasets
- A list of figures that have associated raw data
- A description of any restrictions on data availability

The raw sequence reads and genome assembly will be publicly available on NCBI (https://www.ncbi.nlm.nih.gov) under Project number PRJNA595620 upon publication of the study, and are currently available from the corresponding author on request. The code used for this project is available on Github at https://github.com/kimandrews/Wireworm_popgen

Field-specific reporting

Please select the one below that is the best fit for your research. If you are not sure, read the appropriate sections before making your selection.

☐ Life sciences ☐ Behavioural & social sciences ☒ Ecological, evolutionary & environmental sciences

For a reference copy of the document with all sections, see nature.com/documents/nr-reporting-summary-flat.pdf

Ecological, evolutionary & environmental sciences study design

All studies must disclose on these points even when the disclosure is negative.

Study description	Multiple species and individuals of wireworms (beetle larvae) were collected from multiple sites for population genetic and phylogenetic analysis.
Research sample	Individual wireworms (beetle larvae), primarily from three species, were collected from multiple sites across the northwest US and southwest Canada for population genetic and phylogenetic analysis.
Sampling strategy	Samples were collected using bait traps from multiple sites to examine phylogeographic structure across the northwest US and southwest Canada
Data collection	Multiple researchers collected samples and collected associated metadata. DNA sequencing data was obtained from several sequencing facilities.
Timing and spatial scale	Wireworms were collected using bait traps between 2012 and 2016 during the growing season from April through September of each year.
Data exclusions	No data were excluded.
Reproducibility	Multiple samples were collected from each geographic location over multiple time periods by multiple researchers. Multiple different types of statistical tests were conducted to evaluate population structure.
Randomization	Samples were organized into groups based on species identification and geographic sampling location.
Blinding	During bioinformatic analysis, sample names were used that did not correspond with species names or geographic location when conducting statistical tests that did not require this information, (e.g., sNMF analysis and PCAs)

Did the study involve field work? ☒ Yes ☐ No

Field work, collection and transport

Field conditions	Wireworms (beetle larvae) were collected from agricultural fields using bait traps placed about 15 cm underground.
Location	Wireworms were collected from agricultural fields in the the northwestern US and southwestern Canada (see Fig. 1 and Table S1), using bait traps placed about 15 cm underground.
Access and import/export	Permits were not required for sample collection and export of preserved wireworms.
Disturbance	No disturbance was caused by this research.

Reporting for specific materials, systems and methods

We require information from authors about some types of materials, experimental systems and methods used in many studies. Here, indicate whether each material, system or method listed is relevant to your study. If you are not sure if a list item applies to your research, read the appropriate section before selecting a response.

Materials & experimental systems

n/a	Involved in the study	
☒	☐	Antibodies
☒	☐	Eukaryotic cell lines
☒	☐	Palaeontology
☐	☒	Animals and other organisms
☒	☐	Human research participants
☒	☐	Clinical data

Methods

n/a	Involved in the study	
☒	☐	ChIP-seq
☒	☐	Flow cytometry
☒	☐	MRI-based neuroimaging

Animals and other organisms

Policy information about studies involving animals; ARRIVE guidelines recommended for reporting animal research

Laboratory animals	The study did not involve laboratory animals.
Wild animals	Wireworms (beetle larvae) were collected using bait traps placed about 15 cm underground. Wireworms were preserved by placing in ethanol. Age and sex were unknown.
Field-collected samples	The study did not include laboratory work with live animals collected from the field.
Ethics oversight	No ethical approval was required because working with beetles does not require this.

Note that full information on the approval of the study protocol must also be provided in the manuscript.
